# Enhanced photodynamic therapy through multienzyme-like MOF for cancer treatment

**DOI:** 10.3389/fbioe.2023.1338257

**Published:** 2024-01-19

**Authors:** Letian Lv, Zhao Fu, Qing You, Wei Xiao, Huayi Wang, Chen Wang, Yanlian Yang

**Affiliations:** ^1^ CAS Key Laboratory of Standardization and Measurement for Nanotechnology, CAS Key Laboratory of Biological Effects of Nanomaterials and Nanosafety, CAS Center for Excellence in Nanoscience, National Center for Nanoscience and Technology, Beijing, China; ^2^ University of Chinese Academy of Sciences, Beijing, China

**Keywords:** photodynamic therapy, MOF, nanozyme, multi-functional nanoplatform, combination therapy

## Abstract

Overcoming resistance to apoptosis is a major challenge in cancer therapy. Recent research has shown that manipulating mitochondria, the organelles critical for energy metabolism in tumor cells, can increase the effectiveness of photodynamic therapy and trigger apoptosis in tumor cells. However, there is currently insufficient research and effective methods to exploit mitochondrial damage to induce apoptosis in tumor cells and improve the effectiveness of photodynamic therapy. In this study, we present a novel nanomedicine delivery and therapeutic system called PyroFPSH, which utilizes a nanozymes-modified metal-organic framework as a carrier. PyroFPSH exhibits remarkable multienzyme-like activities, including glutathione peroxidase (GPx) and catalase (CAT) mimicry, allowing it to overcome apoptosis resistance, reduce endogenous glutathione levels, and continuously generate reactive oxygen species (ROS). In addition, PyroFPSH can serve as a carrier for the targeted delivery of sulfasalazine, a drug that can induce mitochondrial depolarization in tumor cells, thereby reducing oxygen consumption and energy supply in the mitochondria of tumor cells and weakening resistance to other synergistic treatment approaches. Our experimental results highlight the potential of PyroFPSH as a versatile nanoplatform in cancer treatment. This study expands the biomedical applications of nanomaterials as platforms and enables the integration of various novel therapeutic strategies to synergistically improve tumor therapy. It deepens our understanding of multienzyme-mimicking active nanocarriers and mitochondrial damage through photodynamic therapy. Future research can further explore the potential of PyroFPSH in clinical cancer treatment and improve its drug loading capacity, biocompatibility and targeting specificity. In summary, PyroFPSH represents a promising therapeutic approach that can provide new insights and possibilities for cancer treatment.

## 1 Introduction

Tumor cells have inherent defense mechanisms that can resist apoptosis triggered by single photodynamic therapy. For example, dihydroorotate dehydrogenase (DHODH) in mitochondria can reduce the damage caused by reactive oxygen species to tumor cell mitochondria (Mao et al., 2021). The presence of multiple defense mechanisms in tumor cells leads to incomplete killing of tumor cells with single treatment strategies, resulting in distant metastasis and tumor recurrence. These strategies can trigger sufficient cell damage through synergistic effects and prevent the recurrence and metastasis of tumors in the same treatment process (Shen et al., 2020; Ruan et al., 2021; Xu et al., 2021; Yang et al., 2023).

**SCHEME 1:**
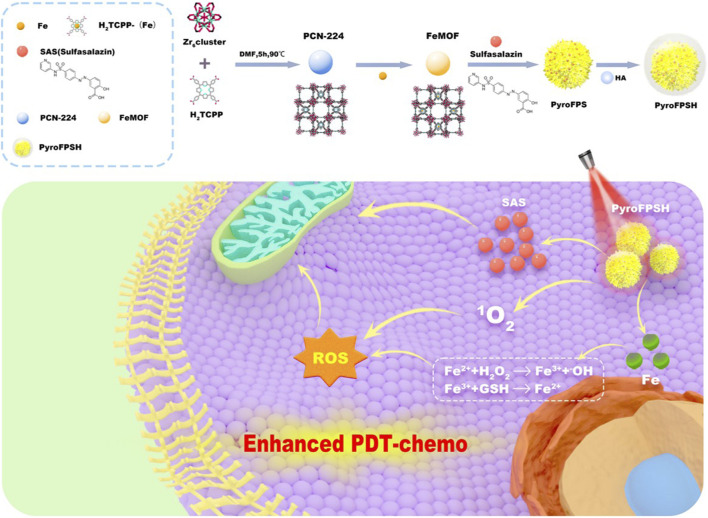
Schematic illustration of the synthesis process of PyroFPSH and its application in tumor treatment. PyroFPSH enhances the efficacy of photodynamic therapy through the combined treatment of nanozymes and small molecule drugs.

The level of cell apoptosis is closely related to levels of ROS arising from singlet oxygen produced by PDT or hydroxyl radicals generated by nanozymes and the corresponding resistance of mitochondria to ROS (Chen et al., 2020; Wan et al., 2020; Chen et al., 2021c; Chen et al., 2022). For nanozymes, several representative nanozymes like Fe_3_O_4_ particles could trigger the decomposition of endogenous H_2_O_2_ into highly reactive hydroxyl (•OH) radicals, invoking an intensive cytotoxic effect on the tumor cells. Building on this knowledge, we developed a strategy involving Fe with diverse nanozyme activities embedded in a photodynamic metal-organic framework PCN-224 to generate Fe@PCN-224 (FeMOF) (Wan et al., 2019; Shi et al., 2020; Chen et al., 2021b). This structure combined nanozyme activity and photodynamic effects as a platform for further modifications with HA and loading of the mitochondrial-targeting drug sulfasalazine (SAS) (Kang et al., 2019; Liu et al., 2021a; Xin et al., 2021). This led to HA@SAS@FeMOF (PyroFPSH) (Scheme 1), which was used to amplify ROS levels, particularly hydroxyl radicals and singlet oxygen, and inhibit malignant tumors. Given the significant reduction in ROS production due to hypoxia, we discovered that Fe embedded in PCN-224 can stimulate the generation of ROS and simultaneously release O_2_ through a chemical kinetic process initiated by the Fenton reaction. Concurrently, the induction of mitochondrial polarization by SAS could further reduce O_2_ consumption and energy production in tumor cell mitochondria. This mitochondrial polarization induced by SAS is thought to alleviate hypoxia, elevate ROS levels, and establish a window period during photodynamic therapy (Jiang et al., 2020; Zhang et al., 2023). Within this SAS-induced window period, severely weakened tumor cells are unable to combat ROS through O_2_-generated energy, thereby minimizing resistance to the synergistic antitumor effects of different therapeutic strategies. Notably, in the presence of a 660 nm laser, PyroFPSH not only stimulates the production of various types of ROS through photodynamic therapy and nanozyme activity, but also enhances the SAS drug release process. In summary, nanozyme catalysis and mitochondrial polarization increase intracellular O_2_ concentrations, thereby alleviating hypoxia. Together with the increase in ROS accumulation in tumors, this leads to the activation of cell death, yielding impressive therapeutic effects in both *in vitro* and *in vivo* antitumor treatments.

Reactive oxygen species (ROS)-induced cell death has been used in various processes including apoptosis, ferroptosis, and pyroptosis (Zhou et al., 2018; Sun et al., 2021; Zhou et al., 2022). Photodynamic processes use a near-infrared laser that can penetrate tumor tissue. When this type of laser is introduced, it can enhance the catalytic activity and therapeutic effect of nanozymes. In particular, the catalytic activity of nanozymes can be enhanced by photodynamic therapy (PDT), whereby a light-augmented Fenton reaction enhances ROS generation under light illumination, significantly increasing the enzymatic activity and the corresponding reaction rate (Chen et al., 2020; Cui et al., 2021; Meng et al., 2021; Hu et al., 2022). Therefore, integrating PDT-enhanced enzymatic activity into a nanozyme can effectively suppress tumor growth and recurrence.

Metal-organic frameworks (MOF) represent a new type of nanomaterials characterized by excellent biocompatibility and a large surface area (Cui et al., 2023). Compared to traditional drug carriers, MOFs can deliver a higher drug dose. Current research in targeted cancer therapy focuses on the development of new materials based on MOFs. By capitalizing on the interaction between the tumor microenvironment (TME) and MOFs, we can develop TME-responsive MOFs to achieve effective TME-targeted cancer treatment. The excellent loading capacity of MOFs makes them effective carriers for photosensitizers, nanozymes, and drugs in tumor therapy.

In this study, we successfully constructed a drug delivery platform, FeMOF@HA, that combines nanozymes with photodynamic therapy (PDT) and chemotherapy (CDT) for synergistic tumor treatment. FeMOF@HA can also deliver the small molecule drug SAS, which regulates mitochondria, reduces tumor cell tolerance to combination therapy, induces tumor cell apoptosis, and accelerates cancer treatment. This study opens a new perspective on nanozyme and photodynamic therapy (PDT) combination treatment based on significant organelle damage in tumor cells, potentially paving the way for new approaches to cancer treatment and innovative strategies for combination cancer therapy.

## 2 Experimental section

### 2.1 Materials and instrumentation

Hydrogen peroxide (H_2_O_2_, 30 wt%) was obtained from Beijing Chemical Reagent Research Institute (Beijing, China). The catalase (3,500 units mg-1 protein from cow liver) was acquired from Beyotime Biotechnology (Shanghai, China). 2,2,6,6-Tetramethylpiperidine (TEMP, 99% pure) was provided by Alfa Aesar. 5,5-Dimethyl-1-pyrroline N-oxide (DMPO, 98% pure) was acquired from Innochem (Beijing, China). TMB (99% pure), 1,3-diphenylisobenzofuran (DPBF, 97% pure), 9,10-anthracenediyl-bis (methylene)dimalonic acid (ABDA, 98% pure) and Tris-HCl (pH 7.4) were obtained from Solarbio. Tetrakis (4-carboxyphenyl) porphyrin (TCPP), hyaluronic acid (HA) (MW, 7.9 kDa), N,N-dimethylformamide (DMF), benzoic acid, zirconium chloride octahydrate (ZrOCl_2_·8H_2_O, 99.99%), and 5,5′-Dithiobis (2-nitrobenzoic acid) (DTNB) were purchased from Aladdin Reagent Co., Ltd. (Shanghai, China). [Ru (dpp)_3_]Cl_2_ (RDPP), glutathione (GSH), 3,3′,5,5′- tetramethylbenzidine (TMB), methylene blue (MB), 2,7-dichlorodihydrofluorescein diacetate (DCFH-DA), and FeCl_3_·6H_2_O were purchased from Sigma-Aldrich. Fetal bovine serum (FBS) and high glucose Dulbecco’s modified Eagle medium (RPMI 1640) were purchased from Gibco Life Technologies. Calcein-AM and propidium iodide (PI) kit, 4′,6-diamidino-2-phenylindole (DAPI), 2,7-dichlorofluorescein diacetate (DCFH-DA), assay kit and JC-1 staining kit were purchased from Beyotime Biotechnology (Shanghai, China).

Topography and elemental mapping images were acquired using Ht-7700 transmission electron microscopy (Hitachi, Japan) and high-resolution TEM (HRTEM) (Tecnai G2 F20, United States), respectively. ICP measurement was performed using the Thermo Scientific iCAP 6,300. The ESR measurements were performed at ambient temperature in a Bruker EMX EPR spectrometer (Billerica, MA). The surface modification was identified by FT-IR spectroscopy (Spectrum One, United States). Zeta potential measurement was performed using the Zetasizer instrument (Zetasizer Nano ZS, England). The chemical composition and crystal structure of the nanosheets were analyzed using XPS (ESCALAB 250Xi, England). UV–Vis spectra were recorded using a Lambda UV–Vis spectrophotometer (Perkin Elmer, United States). Dynamic light scattering (DLS) for particle size and zeta potential was determined using Zetasizer Nano ZS (Malvern, United Kingdom). The XRD patterns were tested on a TZY-XRD (Rigaku, Japan). The experiment of Barrett–Joyner–Halenda model from the adsorption branch of isotherms was performed using an automatic surface area and porosity analyzer (Micromeritics Instrument, United States). The laser of 660 nm was managed by a power-tunable infrared laser (Laserwave, China).

### 2.2 Fabrication of PCN-224 and Fe@PCN-224

PCN-224 were fabricated as reported. In a typical experiment, 100 mg H_2_TCPP, 300 mg ZrOCl_2_·8H_2_O, and 2.8 g benzoic acid were first dissolved in 100 mL DMF. Then, the solution was heated to 90°C for 5 h while stirring (300 rpm). Finally, the PCN- 224 were recovered by centrifugation and washing with DMF three times and stored in fresh DMF for further analysis.

As for Fe@PCN-224, typically, 30 mg of PCN-224 were dispersed in 10 mL DMF containing 40 mg of FeCl_3_. The solution was stirred for 10 min at room temperature and then was heated at 120°C for 7 h with slow magnetic stirring. After the reaction, Fe@PCN-224 were collected by centrifugation and washing three times with DMF and stored in fresh DMF for further characterizations.

### 2.3 Preparation of PyroFPSH

A coprecipitation and stirring method was used to prepare the SAS@FeMOF loaded with arylsulfonamide pyridine. First, 10 mg of FeMOF was dispersed in 10 mL of an ethanol solution. Then, 10 mL of a DMSO solution containing dissolved SAS at a concentration of 1 mg/mL was added. The solution was then stirred overnight at room temperature, protected from light. Next, 5 mL of 1 mg/mL FeMOF and SAS@FeMOF were mixed with 5 mL of hyaluronic acid (HA, Mw: 7.9 kDa) at 1 mg/mL and sonicated for 15 min. The obtained FeMOF@HA and PyroFPSH were collected and further purified by centrifugation. After stirring for 24 h, the nanoparticle was recovered by centrifugation at 1,200 rpm, where the modification of hyaluronic acid was characterized by FTIR. All supernatants were collected by centrifugation to measure the drug loading amount of SAS, and the absorbance value at 356 nm was detected by UV-Vis spectroscope to evaluate the loading capacity of SAS using the following formula:

Loading capacity = (M_SAS_-M_uSAS_)/M_PyroFPSH_×100%, where M_SAS_, M_uSAS_, and M_PyroFPSH_ were the total mass of SAS unloaded SAS and PyroFPSH, respectively.

### 2.4 Enzyme-mimic catalysis

To test the peroxidase-like activity, a solution containing FeMOF@HA (100 μg/mL), TMB (0.5 mM) and H_2_O_2_ (0.5 mM) in acetate buffer (100 mM, pH 4.0) was incubated for 10 min, followed by absorption measurement at 652 nm.

To establish the correlation between GSH consumability and different concentrations of FeMOF@HA, a solution of 10 mM GSH and 20 μM DTNB was introduced into 1 mL of PBS solution. The solution was then centrifuged and the supernatant was subjected to UV-Vis spectroscopy for further examination.

### 2.5 Photodynamic activity test

The photodynamic activity was examined in the assay by DPBF or ABDA. For the former, 1 mL of DPBF solution (20 μg/mL) and 100 μL of FeMOF@HA dispersion (100 μg/mL) were mixed, followed by stirring in the dark for 2 h. A continuous semiconductor diode laser (660 nm) with a power density of 50 mW/cm^2^ was then used as a light source for 5 min. Then, the samples were taken for UV-Vis measurements.

For the latter, 1 mL of ABDA solution (100 μg/mL) was mixed with 100 μL of FeMOF@HA (100 μg/mL). A continuous semiconductor diode laser (660 nm) with a power density of 50 mW/cm^2^ was then used as a light source for 5 min. Then, the samples were taken for UV-Vis measurements.

### 2.6 •OH generation by FeMOF@HA

To evaluate the ability of FeMOF@HA to produce •OH, FeMOF@HA samples at different concentrations were mixed with DMPO. The mixture was then exposed to irradiation (660 nm, 100 mW/cm^2^) for 5 min to assess the formation of •OH. The •OH formation served as an indicator of the nitrogen-trapping agent’s efficacy.

### 2.7 GSH reduction–mediated ^1^O_2_ generation of FeMOF@HA

Different concentrations of GSH (0 mM and 10 mM) were prepared and reacted with FeMOF@HA (100 μg/mL) under different pH conditions (pH 7.4 and pH 5.5). The production of ^1^O_2_ was measured using ESR. For the reaction, 100 μL of H_2_O_2_ (10 mM), 600 μL of the reaction mixture, and 300 μL of PBS were mixed. The TEMP scavenger was added to capture ^1^O_2_.

### 2.8 Cell viability assay

EMT-6 cells were planted in 96-well plates at a density of approximately 8 × 10^3^ cells per well and incubated for 24 h, then incubated with different materials (FeMOF@HA, PyroFPSH, FeMOF@HA + Laser, PyroFPSH + Laser) for 24 h. After washing with PBS, the cells were irradiated with a 660 nm laser (50 mW/cm^2^, 5 min). Cell viability was measured 24 h after laser irradiation. Cell viability was assessed using the crystal violet colorimetric assay. At the end of the incubation period, cells were washed with PBS and fixed CCK-8 to determine cell viability.

### 2.9 Detection of O_2_ production

The O_2_ generating ability of FeMOF@HA and PyroFPSH *in vitro* was evaluated using [Ru (dpp)_3_]Cl_2_. EMT-6 cells were incubated in a plate overnight. Then, 100 μL of [Ru (dpp)_3_]Cl_2_ (50 μM) PBS solution was added to the cells and incubated for another 4 h. Then, 0.6 mL of PBS solution containing 100 μg/mL FeMOF@HA and PyroFPSH was added to each well. The cells were then incubated for varying lengths of time, with or without a laser (660 nm, 50 mW/cm^2^). Finally, the cells were observed using CLSM.

### 2.10 Intracellular ROS assay

EMT-6 cells (6 × 10^3^ cells/well) were seeded in a 96-well plate and incubated for 12 h. The medium was replaced with complete RPMI 1640 medium containing different materials (FeMOF@HA, PyroFPSH, FeMOF@HA + Laser, PyroFPSH + Laser) at a dose of 100 μg/mL for 12 h. After washing with PBS, the cells were irradiated with a 660 nm laser (50 mW/cm^2^, 5 min). The DCFH-DA was added following the standard protocol provided by the supplier. After 20 min of incubation, the cells were washed twice with PBS and the fluorescence images of the treated cells were captured under an inverted fluorescence microscope.

### 2.11 Establishment of orthotopic 4T1 tumor models

BALB/c mice (female, 6–8 weeks old) were obtained from Beijing Vital River Experimental Animal Technology Co. Ltd. All animal experiments were approved by the Institutional Animal Care and Use Committee of the Chinese Academy of Medical Sciences Institute of Tumors (NCNST21-2108-0610). All procedures were performed according to guidelines. To establish the tumor models, 2 × 10^6^ EMT-6 cancer cells suspended in PBS were implanted subcutaneously into the right mammary fat pad of each mouse as primary tumors.

### 2.12 *In Vivo* therapeutic experiment

When the tumor size reached about 80 mm^3^, the EMT-6 tumor-bearing BALB/c mice were randomly divided into eight groups (*n* = 5) and injected intratumorally with saline, FeMOF@HA, PyroFPSH (FeMOF@HA dose: 100 μg injected/mouse). After 12 h of injection, the mice were anesthetized with 2% (v/v) isoflurane and the tumors were irradiated with light (660 nm, 100 mW/cm^2^, 5 min). Tumor sizes were measured daily with a caliper, with tumor volume equal to (width^2^ × length)/2. The body weight of each group was monitored every 2 days. The mice were euthanized on day 14 and the excised tumors were photographed and weighed. Hematological and Histological Analysis.

Blood was collected from each mouse at day 14 and centrifuged at 2000 *g* for 15 min and the separated serum samples were tested for blood biochemical markers. The major organs (heart, liver, spleen, lung, and kidney) and the tumor were dissected and sectioned for TUNEL and H&E staining.

### 2.13 Statistical analyses

All results were presented as mean ± S.D. Means were indicated using the Student’s t-test. Statistical significance was determined at a value of *p < 0.05, **p < 0.01, ***p < 0.001.

## 3 Results

### 3.1 Synthesis and characterization of the PyroFPSH

**FIGURE 1:**
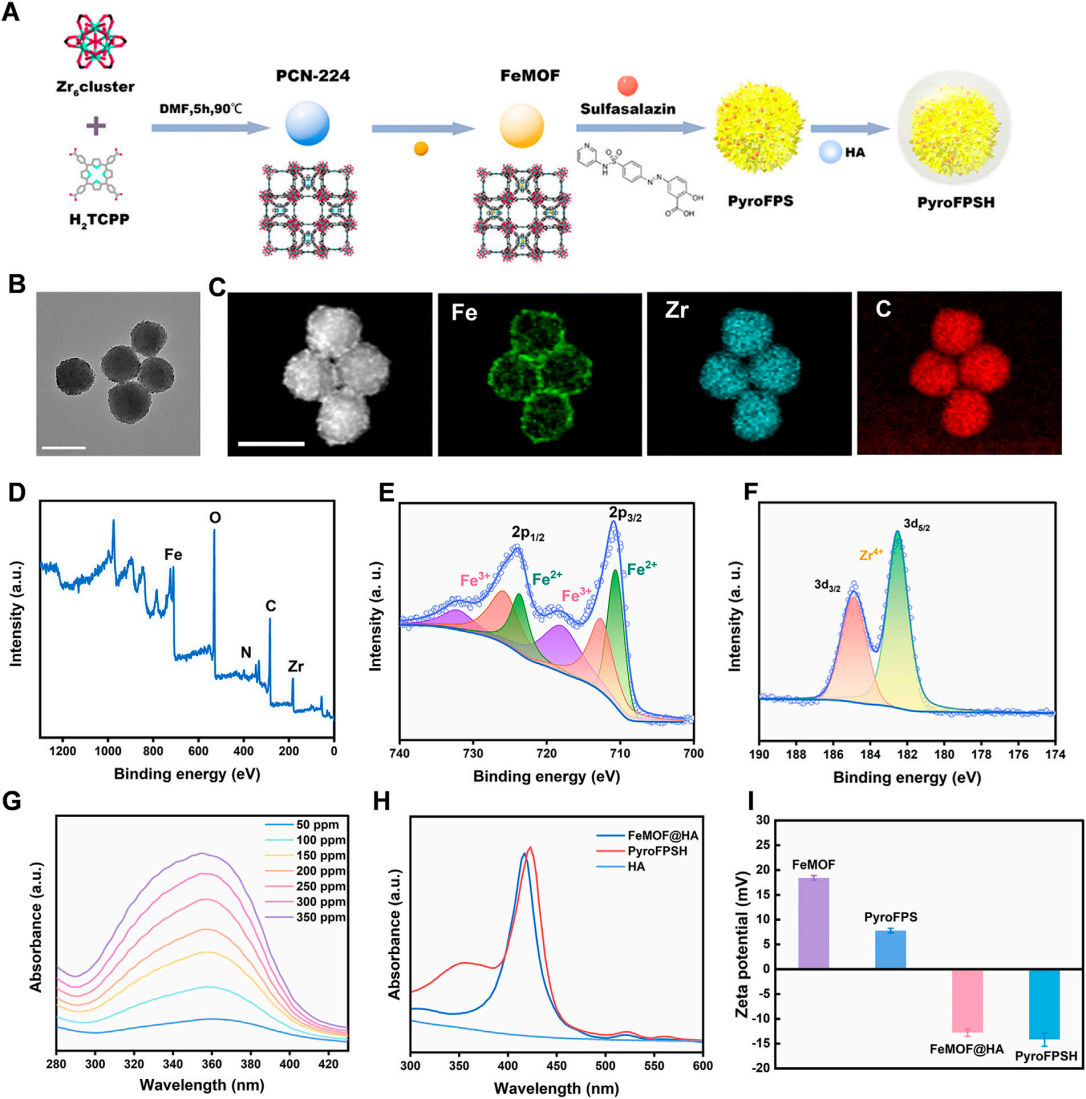
Synthesis and characterization of PyroFPSH. **(A)** The schematic of the synthetic preparation of FeMOF. **(B)** TEM image of FeMOF (scale bar: 100 nm). **(C)** HAADF-STEM image and elemental mapping of FeMOF (scale bar: 100 nm). **(D)** XPS spectrum of FeMOF. **(E)** High-resolution Fe 2p XPS spectrum of FeMOF. **(F)** High-resolution Zr 3d XPS spectrum of FeMOF. **(G)** UV-visible absorption spectra of sulfasalazine at different concentrations. **(H)** The UV-Vis absorption spectra of various samples including HA, FeMOF@HA, and PyroFPSH. **(I)** The zeta potential of different samples including FeMOF@HA, PyroFPS, FeMOF@HA, and PyroFPSH.

The synthesis of Fe@PCN-224 began with the preparation of PCN-224 using a well-established method (Figure 1A). Subsequently, Fe^3+^ was doped into the PCN-224 at 120°C for 8 h, resulting in the production of Fe@PCN-224 (Ding et al., 2020b). Once the reaction between Fe^3+^ and PCN-224 occurred, the resulting Fe@PCN-224, also known as FeMOF (Figure 1B), retained the same size and morphology as the original PCN-224 (Wang et al., 2021; Zhu et al., 2023). Additionally, STEM mapping images and energy dispersive spectroscopy (EDS) showed a uniform distribution of C, O, N, Zr, and Fe elements, confirming the successful synthesis of FeMOF (Supplementary Figure S1). The successful preparation of Fe@MOF was confirmed by energy dispersive X-ray spectrometer (EDS) analysis and the corresponding elemental mapping, which showed the coexistence of the elements Fe and Zr (Figure 1C). Further confirmation of the successful modification was obtained by XPS analysis performed to determine the chemical state of the Fe species in the Fe@MOF. The presence of Fe^3+^ was indicated by a peak at 711 eV in the Fe 2p core level spectrum (Figures 1D–F).

Sulfasalazine (SAS) is an FDA-approved small molecule drug. Research has shown that SAS can induce the accumulation of mitochondrial peroxides in tumor cells by weakening mitochondria’s tolerance to reactive oxygen species (ROS). In this study, we developed a nanodrug delivery system, SAS@Fe@PCN-224 (PyroFPS), by co-incubating Fe@PCN-224 (FeMOF) with SAS. To improve the stability and dispersibility of SAS@FeMOF under physiological conditions, we further modified it with hyaluronic acid, resulting in HA-SAS@FeMOF (PyroFPSH) (Supplementary Figure S2). By comparing the observable differences in properties (like DLS and Zeta potential) of PyroFPSH with the original FeMOF before and after drug loading (Supplementary Figures S3–S5), we further confirm the successful HA coating and successful SAS loading (Figures 1G, H). When negatively charged sulfasalazine was loaded onto FeMOF, the surface potential changed from positive (18.6 mV) to negative (−12.4 mV). Compared to the positive potential of PyroFPS (8.28 mV), the potential of PyroFPSH decreases to −12.4 mV (Figure 1I), indicating that HA was successfully coated (Kim et al., 2019; Ding et al., 2020a).

### 3.2 Multienzyme activities of FeMOF@HA

Given the significant relationship between enzyme activity and anticancer properties, our investigation focused on whether FeMOF@HA has multienzyme-like activity (Chen et al., 2021a). The literature suggests that Fe ions, due to their peroxidase-like activity, can exploit the presence of H_2_O_2_ in tumor cells to generate significant hydroxyl radicals through the Fenton reaction. These radicals, in conjunction with the singlet oxygen generated during photodynamic therapy, cause oxidative damage to key intracellular organelles. To counteract this, tumor cells synthesize large amounts of GSH to compensate for oxidative damage and limit their susceptibility. Additionally, the Fe^3+^ generated from the H_2_O_2_ reaction interacts with the abundant intracellular GSH, attenuating the protective mechanism of tumor cells and augmenting the synergistic therapeutic effects of FeMOF@HA under diverse treatment regimens. Therefore, iron-based FeMOF@HA are an integral part of nanocatalytic therapy.

**FIGURE 2:**
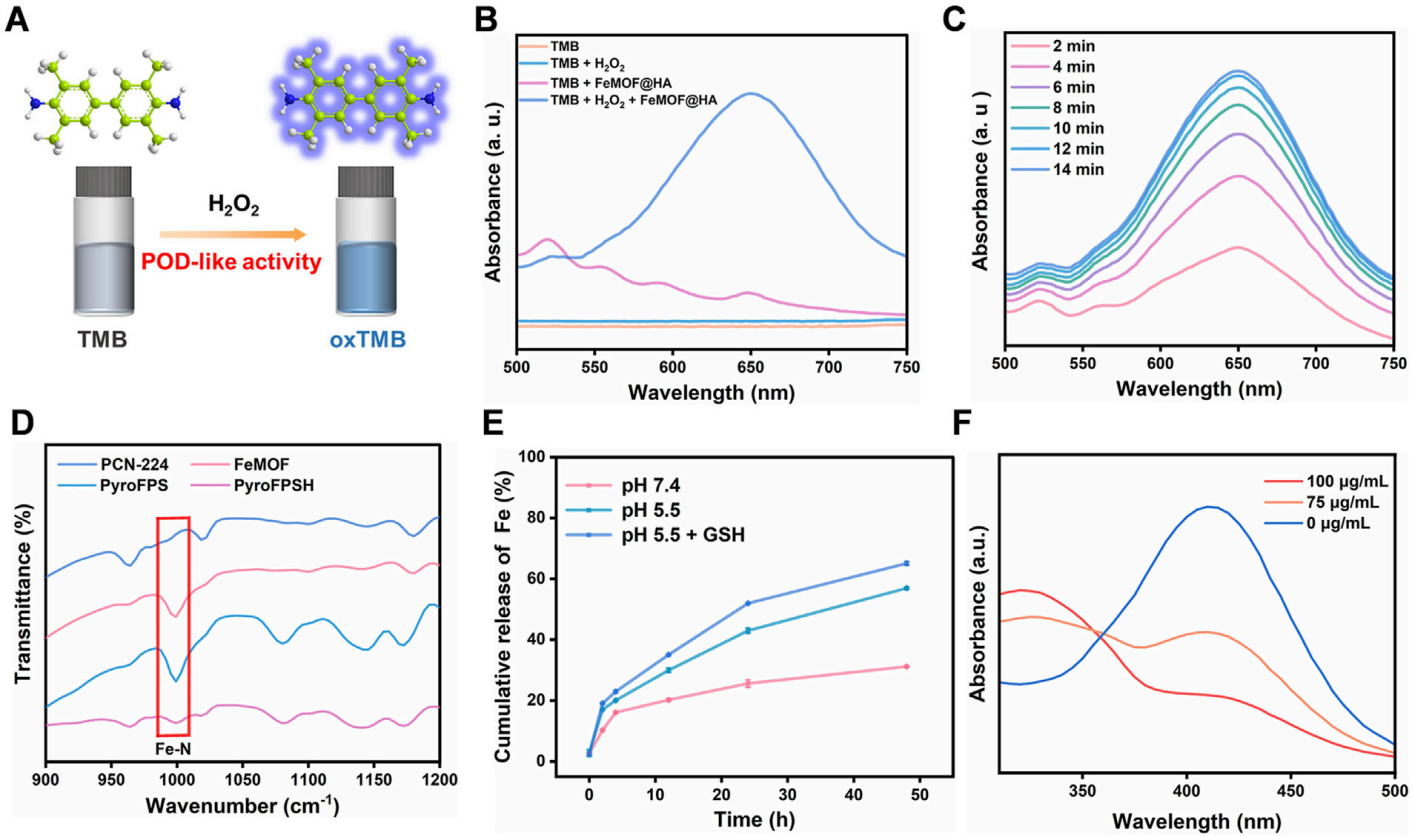
**(A)** The schematic diagram of the POD-like catalytic process of FeMOF@HA. **(B)** UV absorption spectra of the mixture of FeMOF@HA (100 μg/mL), TMB (0.5 mM), and H_2_O_2_ (0.5 mM). **(C)** Time-dependent changes in the absorption spectra of the mixture of FeMOF@HA (100 μg/mL), TMB (0.5 mM), and H_2_O_2_ (0.5 mM). **(D)** FT-IR spectroscopy of Fe-N. **(E)** The quantitative analysis of Fe release from FeMOF@HA (100 μg/mL), in different microenvironments (pH 7.4, pH 5.5, and pH 5.5 + GSH). Data represent the mean ± SD of 3 independent experiments. **(F)** UV–vis absorption spectra of DTNB (25 μg/mL) incubated with GSH (100 μM) plus FeMOF@HA.

FeMOF@HA follow a definitive catalytic therapeutic mechanism. In the slightly alkaline tumor cell environment, Fe^3+^ in FeMOF@HA readily reacts with intracellular H_2_O_2_ to form O_2_ and Fe^2+^. The electrode redox potential for this reaction (Fe^3+^ to Fe^2+^ [φθ(Fe^3+^/Fe^2+^ = 0.77 V)]) is significantly higher than that of H_2_O_2_ [φθ(O_2_/H_2_O_2_ = 0.68 V)] and further substantiates the peroxidase-like (POD) activity of FeMOF@HA with the oxidation of tetramethylbenzidine (TMB) upon introduction of H_2_O_2_ (Figure 2A) (Li et al., 2019; Liang et al., 2021). This reaction yields a blue-green product with a peak absorption at 652 nm, confirming the peroxidase-like behavior of FeMOF@HA (Figures 2B, C). In summary, the multienzyme-like activity of FeMOF@HA allows it to neutralize ROS in tumor cells and enhance the efficacy of photodynamic therapy, resulting in improved antitumor treatment (Supplementary Figure S5).

Furthermore, FT-IR spectroscopy was used to analyze the incorporation of Fe^3+^. The results showed that the asymmetric vibration absorption intensity of C=O and C-OH of both PCN-224 and Fe@MOF was significantly lower than that of H_2_TCPP. This is attributed to the coordination between the Zr6 cluster and the carboxyl groups. Further evidence for Fe^3+^ coordination with porphyrin was provided by the symmetrical Fe-N stretching observed at approximately 1,000 cm^−1^ (Figure 2D). ICP-MS analysis revealed that the weight percentage of Fe in Fe@PCN-224 was 15%.

As illustrated in Figure 2E, the release of Fe ions remains minimal in the absence GSH, indicating the stable nature of FeMOF@HA. However, when FeMOF@HA is exposed to GSH, it triggers a sustained release of Fe ions (Liu et al., 2021b). FeMOF@HA not only mimics peroxidase and photosensitization activity, but also can deplete glutathione in tumor cells, thus amplifying ROS production and augmenting toxicity to tumor cells.

Tumor cells can often negate the effectiveness of ROS-based treatments by upregulating GSH expression. To investigate whether FeMOF@HA can counteract this mechanism, we used 5,5′-dinitrobenzoic acid (2-nitrobenzoic acid) dithioester (DTNB) to monitor GSH levels (Lin et al., 2018; Hu et al., 2019; Yi et al., 2021). DTNB serves as a reliable probe for the thiol group (-SH) in GSH and forms a compound with GSH that has a notable peak at 412 nm. Reduced absorption intensity would indicate GSH depletion. Consequently, FeMOF@HA was co-incubated with a 10 mM GSH solution, with the residual GSH being quantified at different incubation intervals using DTNB. The data in Figure 2F show that at a FeMOF@HA concentration of 100 μg/mL, the absorption peak at 412 nm disappears and only the DTNB absorption peak at 323 nm remains. This outcome supports the notion that FeMOF@HA consumes the ROS scavenger GSH and thereby maintains the ROS levels within cancer cells.

By exploiting their multienzyme-mimicking catalytic activity and GSH-depleting activity, FeMOF@HA could potentially enable efficacious nanocatalytic therapy. These unique properties position FeMOF@HA as a promising therapeutic approach with significant implications for future tumor treatment. Despite these encouraging findings, further research is required to verify the therapeutic efficacy and safety and to optimize the design of the drug delivery system.

### 3.3 Multienzyme activities of the FeMOF@HA

**FIGURE 3:**
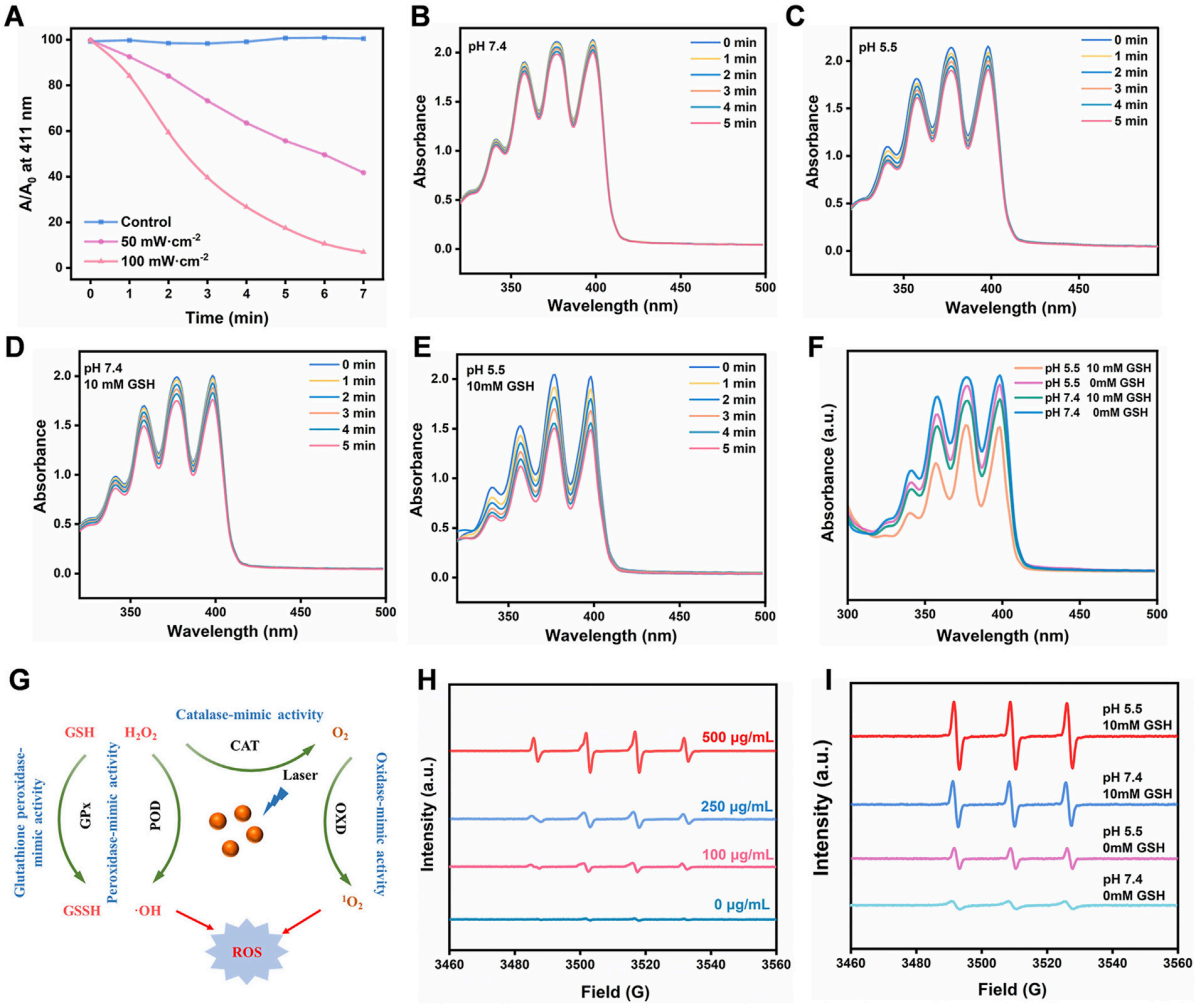
**(A)** Absorbance of DPBF solution (20 μg/mL) under 660 nm laser irradiation at different power levels. **(B–F)** Evaluation of the efficiency of singlet oxygen generation by FeMOF@HA under varying pH and GSH concentration conditions irradiated with a 660 nm laser at a power of 50 mW/cm^2^, assessed by the photovoltaic changes in the signature peak absorbance (379 nm) of ABDA (100 μg/mL). **(G)** The overall multienzyme-like activity mechanism of FeMOF@HA. **(H)** The ESR spectra of •OH in the presence of DMPO. **(I)** The ESR spectra of ^1^O_2_ in the presence of TEMP.

We began our study by using ROS indicators such as 1,3-diphenylisobenzofuran (DPBF) and 9,10-anthracene-bis (methylenemalonic acid) (ABDA) to evaluate the capacity of reactive oxygen species (ROS) generation under light exposure (Shi et al., 2020; Wang et al., 2020). Figure 3A illustrates that the generated ROS can undergo redox reactions with DPBF, as evidenced by a significant decrease in the UV-visible absorption peak at 411 nm. Notably, under 660 nm laser irradiation conditions of 100 mW/cm^2^ and 50 mW/cm^2^ for a duration of 5 min, the absorption intensity of FeMOF@HA rapidly decreased to 26% and 63% of the original value, respectively. This observation highlights the promising potential of FeMOF@HA for photodynamic therapy (PDT).

We first sought to evaluate the ability of FeMOF@HA to produce singlet oxygen, a key component for the effectiveness of photodynamic therapy. The FeMOF@HA were exposed to a laser power of 50 mW/cm^2^ for 5 min, with generation of singlet oxygen leading to changes in ABDA UV absorption from 350–450 nm. Initial experiments without glutathione (GSH) showed minimal effects on photodynamic efficiency, with FeMOF@HA remaining largely unchanged under 5 min of 660 nm laser irradiation (50 mW/cm^2^) and varying pH (Figures 3B, C). In summary, GSH concentration and pH had little influence on the photodynamic efficiency of FeMOF@HA. Subsequently, to further investigate how the photodynamic efficiency of FeMOF@HA is affected by high GSH concentrations, we simulated normal and tumor microenvironmental conditions. In contrast to normal conditions, the absorbance of FeMOF@HA decreased much faster under tumor conditions when exposed to the same 660 nm laser dose (Figures 3D, E). This enhanced absorption decrease signifies improved photodynamic efficiency specific to the tumor microenvironment. Overall, the increased photodynamic therapy activity can be attributed to FeMOF@HA in tumor environments (Figure 3F).

In addition, as shown in Figure 3G, the generation of •OH and ^1^O_2_ was quantitatively evaluated using electron spin resonance (ESR) measurement, in which •OH was trapped by 5,5-dimethyl-1-pyrroline-N-oxide (DMPO) (Yang et al., 2020), and 2,2,6,6-tetramethylpiperidine (TEMP) was used to capture ^1^O_2_ (Wu et al., 2022). As the concentration of FeMOF@HA nanoparticles increased, the ESR signal steadily improved, and a stronger ESR signal of ^1^O_2_ from FeMOF@HA was observed compared to other groups under 660 nm laser irradiation at pH 5.5 with 10 mM GSH (Figures 3H, I). These results suggest that the acidic conditions and high GSH concentrations in the tumor microenvironment could activate the fluorescence and PDT of the quenched FeMOF@HA, thereby minimizing the potential damage to normal cells by the photosensitizers.

In summary, our results clearly demonstrate that FeMOF@HA is able to effectively generate ROS, including ·OH and ^1^O_2_, under light exposure, providing promising outcomes for photodynamic therapy.

### 3.4 Validation of the multienzyme-like activities

**FIGURE 4:**
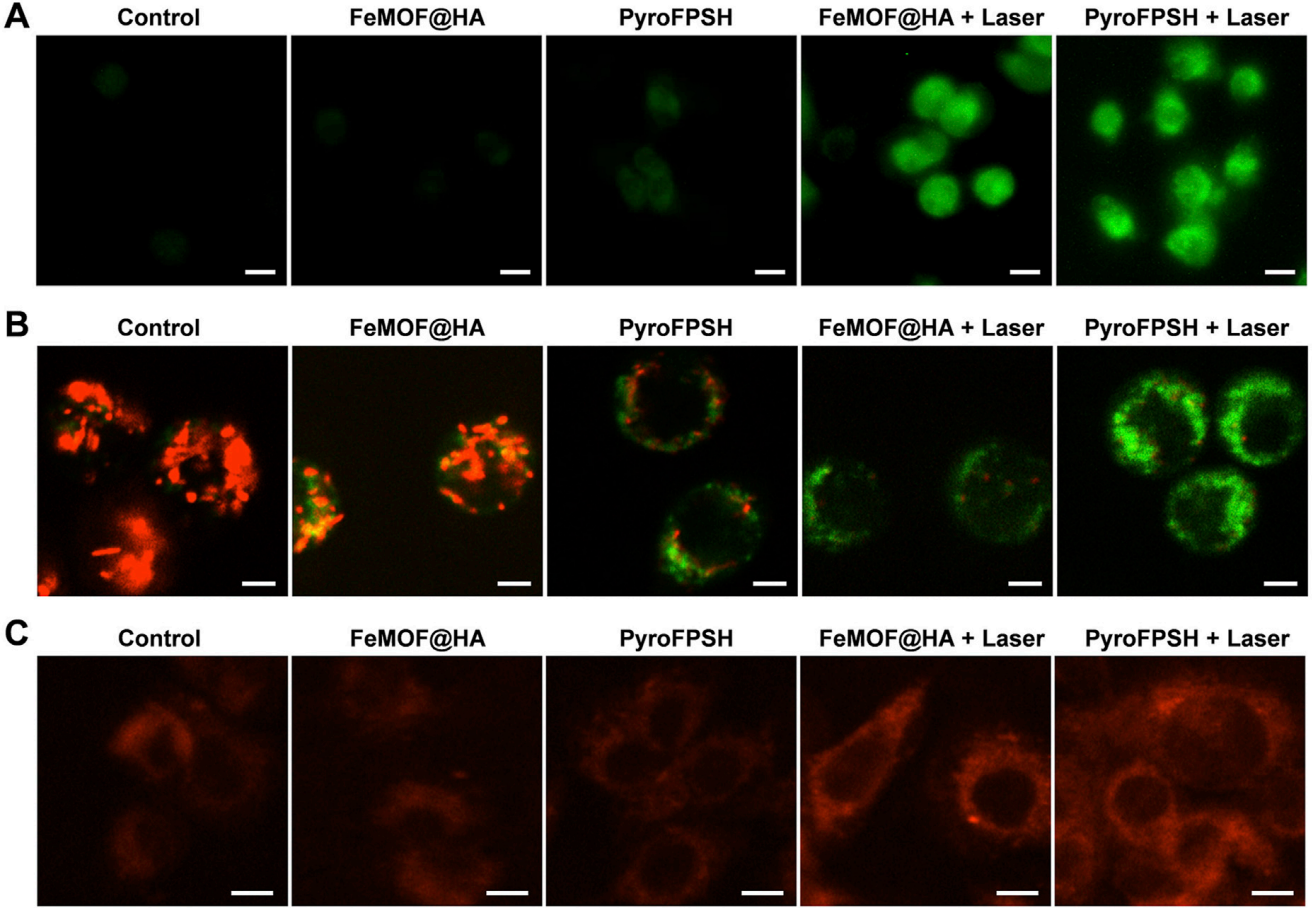
**(A)** CLSM images of EMT-6 cells stained with the ROS probe DCFH-DA under different treatments (scale bar: 20 μm). **(B)** JC-1 assay of EMT-6 tumor cells after coincubation with FeMOF@HA and PyroFPSH across different treatments (scale bar: 10 μm). **(C)** CLSM images of EMT-6 cells stained with O_2_ probe RDPP under different treatments (scale bar: 10 μm).

To evaluate the ability of FeMOF@HA and PyroFPSH to generate reactive oxygen species (ROS) in cells upon exposure to light, we used 2′,7′-dichlorodihydrofluorescein diacetate (DCFH-DA), a ROS probe that emits green fluorescence in the presence of ROS (Li et al., 2021; Wang et al., 2023). This feature was utilized in our investigations using confocal laser scanning microscopy (CLSM). As shown in Figure 4A, we made several interesting observations. Notably, groups treated with FeMOF@HA and PyroFPSH alone without laser exposure exhibited very weak fluorescence. However, when EMT-6 cells cultured with either FeMOF@HA or PyroFPSH were exposed to a 660 nm laser for 5 min, prominent green fluorescence was observed. This suggests that these nanoparticles produce only trace amounts of ROS without laser irradiation, but significantly enhance intracellular ROS levels upon irradiation. Therefore, under laser irradiation conditions, both FeMOF@HA and PyroFPSH show remarkable ROS-generating properties and potential for photodynamic therapy (PDT) applications.

Since SAS targets the mitochondria of tumor cells (Supplementary Figure S7), its mechanism of action involves reducing the resistance of tumor cells to ROS, thereby inducing mitochondrial polarization and tumor cell death (Bao et al., 2021). We hypothesized that the enhanced photodynamic effects of PyroFPSH and increased intracellular ROS levels are related to SAS-induced mitochondrial polarization. Our results showed that PyroFPSH induced the polarization of mitochondrial polarization more noticeably compared to FeMOF@HA under the same conditions, demonstrating the important role of SAS (Figure 4B).

We also investigated the catalysis of O_2_ production by FeMOF@HA and PyroFPSH in EMT-6 cells using [Ru (dpp)_3_]Cl_2_ (RDPP) as an indicator (Hu et al., 2021). In tumor cells, the H_2_O_2_ concentration can be as high as 100 μM, which is significantly higher than in normal cells (below 20 nM). Therefore, an experiment using the O_2_ probe RDPP was performed in live EMT-6 cells to confirm H_2_O_2_-triggered O_2_ production. As shown in Figure 4C, the fluorescence intensity in the groups treated with FeMOF@HA and PyroFPSH is significantly lower than in the control group because the RDPP fluorescence can be quenched by O_2_. When cells were treated with FeMOF@HA + Laser and PyroFPSH + Laser, the fluorescence intensity in the cells showed no significant change compared to the control group (Figure 4C). This clearly indicates that Fe^3+^ in FeMOF@HA and PyroFPSH catalyzes the production of O_2_ within cells triggered by H_2_O_2_ and exhibits catalase-like enzyme activity. This not only overcomes the hypoxia of tumors but also facilitates the production of ^1^O_2_ by PDT.

### 3.5 *In Vitro* therapeutic assessment at the cellular level

**FIGURE 5:**
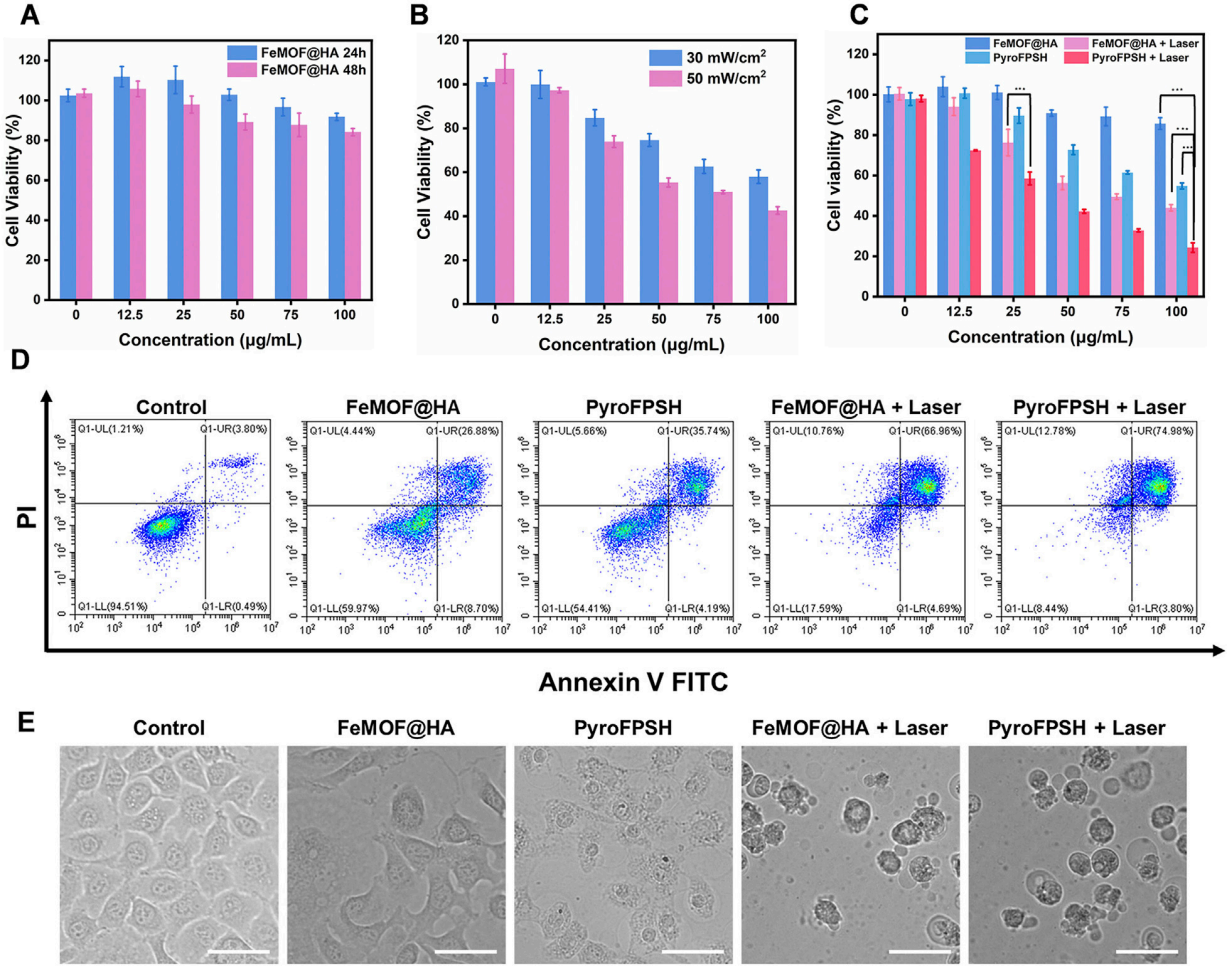
**(A)** Cell viability of EMT-6 cells incubated with FeMOF@HA for 24 h and 48 h at different concentrations. **(B)** Cell viability of EMT-6 cells coincubated with FeMOF@HA under 660 nm laser irradiation at power levels of 30 mW/cm^2^ and 50 mW/cm^2^. **(C)** Cell viability of EMT-6 cells was assessed 48 h post treatment with FeMOF@HA and PyroFPSH. The treatments were carried out with or without laser irradiation (660 nm, 50 mW/cm^2^, 5 min). **(D)** Flow cytometry analysis of apoptosis of EMT-6 cells after different treatments. **(E)** Representative microscopy images (bright-field) of EMT-6 cancer cells after the different treatments. (Scale bar: 50 μm). Data are presented as the mean ± SD. Statistical analysis was performed using the Student’s t-test (ns, nonsignificant. *p < 0.05, **p < 0.01, and ***p < 0.001).

Considering the multienzyme-like activity and photodynamic therapy (PDT) effects of FeMOF@HA and PyroFPSH, we used Cell Counting Kit-8 (CCK-8) to examine the viability of EMT-6 cells under different treatment conditions (Figure 5A). First, we investigated the cytotoxicity of FeMOF@HA nanoparticles as a base carrier for EMT-6 cells. The results showed that FeMOF@HA had relatively low cytotoxicity. We then exposed EMT-6 tumor cells to different laser irradiation conditions (30 mW/cm^2^ and 50 mW/cm^2^) after treatment with 100 μg/mL FeMOF@HA. Cell viability decreased to 57.97% (660 nm, 30 mW/cm^2^) and 42.63% (660 nm, 50 mW/cm^2^), respectively (Figure 5B).

To assess the effect of SAS, we further examined its effects on cell viability (Figure 5C). EMT-6 cancer cells were coincubated with different concentrations of FeMOF@HA and PyroFPSH (0, 12.5, 25, 50, 75, and 100 μg/mL) for 48 h, followed by laser irradiation at 660 nm, 50 mW/cm^2^ for 5 min. The activity of EMT-6 cells significantly decreased with increasing PyroFPSH concentration, suggesting that the addition of SAS can enhance the photodynamic therapy effect of PyroFPSH + Laser. The flow cytometry image (Figure 5D) showed varying degrees of apoptosis in the different treatment groups, which is consistent with the results of the CCK-8 cytotoxicity assay.

Furthermore, the morphology of FeMOF@HA-treated cells showed no significant changes. However, treatment with FeMOF@HA + Laser, PyroFPSH, and PyroFPSH + Laser resulted in the appearance of obvious balloon-like membrane protrusions in EMT-6 cells (Figure 5E), which are characteristic features of cell death.

### 3.6 *In Vivo* antitumor effect

**FIGURE 6:**
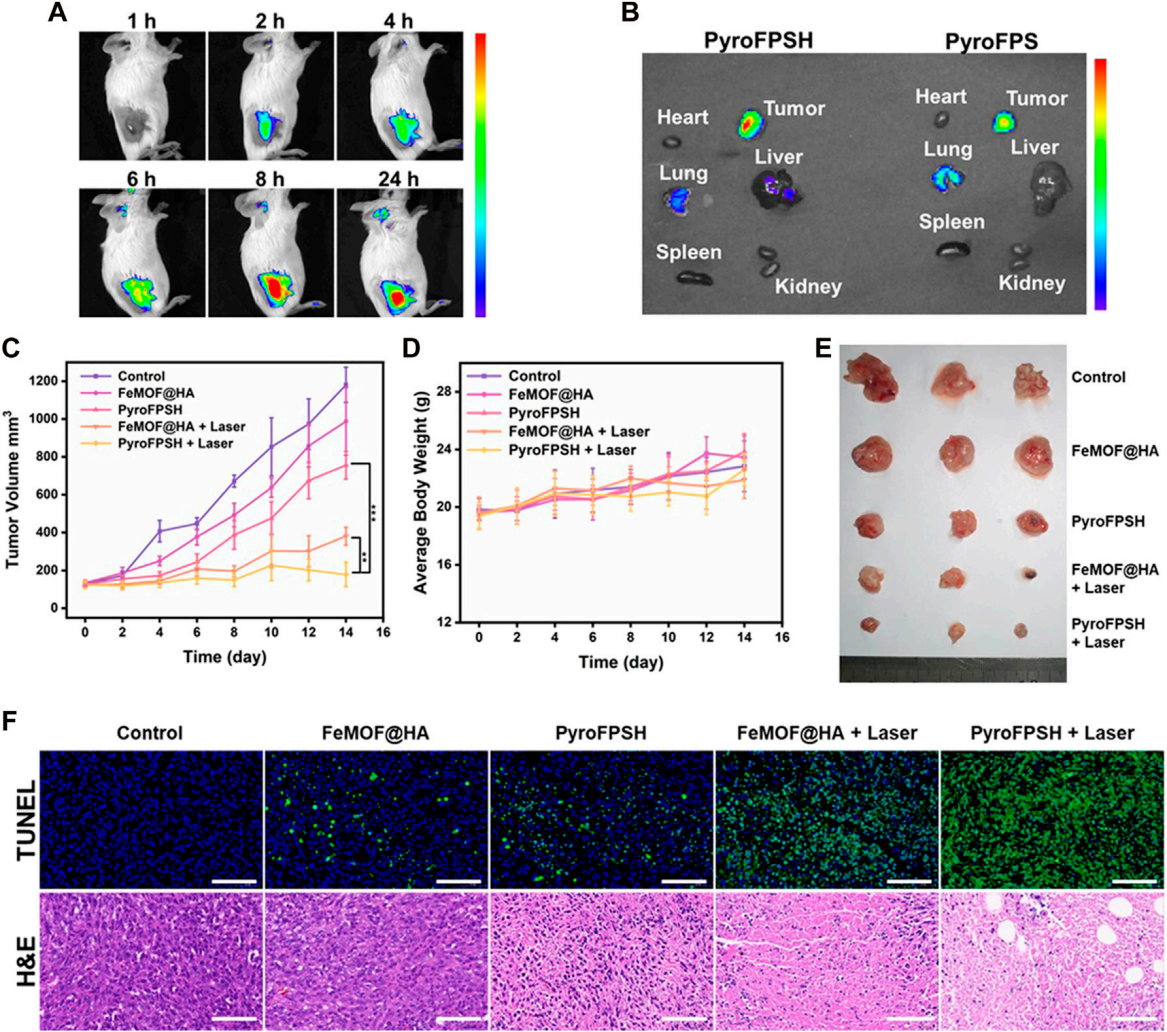
**(A)** FL imaging of EMT-6 tumor-bearing mice injected with PyroFPSH through the tail vein at different time points. **(B)** FL imaging of major organs and tumors 24 h post injection. **(C)** Schematic representation of the establishment and treatment of the EMT-6 tumor-bearing BALB/c mouse model. **(D)** Body weight of mice during 14 days of treatment. **(E)** Images of tumors after 14 days of treatment. **(F)** The representative H&E staining and TUNEL staining images of tumors with different treatments (scale bar: 50 μm). Data are presented as the mean ± SD. Statistical analysis was performed using one-way ANOVA.

We monitored the biodistribution of various components of nanoparticles and observed effective accumulation of FeMOF@HA at the tumor site after intravenous injection, which exhibited strong fluorescence signals (Figure 6A). Additionally, Figure 6B showed that HA with a lower relative molecular weight increased the accumulation of nanoparticles at the tumor site while reducing the accumulation in the lung, indicating the tumor-targeting effect of HA *in vivo*.

Given the positive results obtained with FeMOF@HA and PyroFPSH in terms of catalytic, photodynamic, and cellular therapeutic effects, we evaluated their antitumor efficacy *in vivo*. A breast cancer model was established by subcutaneous injection of EMT-6 cells into female BALB/c mice at 3–4 weeks of age. The tumor-bearing mice were randomly divided into five groups (n = 5 per group): 1) control group; 2) FeMOF@HA group; 3) PyroFPSH group; 4) FeMOF@HA + Laser group; 5) PyroFPSH + Laser group (intravenous injection, 2 mg/kg). Each group received a tail vein injection and corresponding treatment every 12 h, with photodynamic therapy (660 nm, 100 mW/cm^2^, 5 min) applied to the respective components on days 1, 3, and 5.

Figure 6C shows that mice treated with free FeMOF@HA, PyroFPSH, FeMOF@HA + Laser, and PyroFPSH + Laser exhibited tumor inhibition effects compared to the control group, with the PyroFPSH + Laser group demonstrating the strongest antitumor effects. The body weight and tumor volume of the mice were monitored every 2 days throughout the treatment period. Importantly, no significant changes in body weight were observed in any of the groups, indicating the high biosafety of the treatment (Figure 6D).

The final tumor volume and quality of excised tumors were consistent with the antitumor effects observed in different experimental groups (Figure 6E). To further investigate the therapeutic effects of the materials on tumor-bearing mice, tumor tissue sections from each group were stained with H&E and TUNEL, as shown in Figure 6F. In the five experimental groups, the rate of cell necrosis within the tumor tissue increased with increasing treatment intensity, indicating the therapeutic effects of the nano-drug delivery system. In addition, compared to the control group, the results of hematoxylin-eosin (H&E) staining of major organs (heart, liver, lungs, spleen, and kidneys) in mice (Supplementary Figure S8), as well as serum factor analysis, showed no significant pathological abnormalities or inflammatory lesions in the treatment group, confirming the excellent tissue compatibility of the materials (Supplementary Figure S9).

## 4 Conclusion

In conclusion, we developed PyroFPSH as a platform to synergize photodynamic therapy with multiple enzyme-mimicking activities for improved antitumor efficacy. The PyroFPSH integrate FeMOF@HA for enzyme-like activities, including peroxidase (POD), oxidase (OXD) to generate reactive oxygen species (ROS), and catalase (CAT) to counteract hypoxia. In addition, PyroFPSH contains SAS to induce mitochondrial dysfunction in tumor cells and reduce their metabolic activity and resistance to treatment. Mechanistically, SAS induces mitochondrial polarization in tumor cells through PyroFPSH, while photodynamic therapy simultaneously generates ROS. This therapeutic window of mitochondrial polarization and elevated ROS results in potent cytotoxicity. Evaluations in an EMT-6 tumor model showed a significant inhibition of tumor growth. Overall, PyroFPSH can effectively induce tumor cell death by combining nanozyme activity with photodynamic and SAS combination therapy thereby presenting a promising new approach for cancer treatment.

## Data Availability

The original contributions presented in the study are included in the article/Supplementary Material, further inquiries can be directed to the corresponding authors.
