# Supplementary material for: Enhanced photodynamic therapy through multienzyme-like MOF for cancer treatment
Source: Front Bioeng Biotechnol. 2024 Jan 19;11:1338257. doi: 10.3389/fbioe.2023.1338257 (PMC10834778; doi:10.3389/fbioe.2023.1338257)
Supplement: Supplementary file 1 [file DataSheet1.docx]

Supplementary Material

Enhanced Photodynamic Therapy Through Multienzyme-like MOF

for Cancer Treatment

Letian Lv^1,2^, Zhao Fu^1^, Qing You^1^, Wei Xiao^1^, Huayi Wang^1^, Chen Wang^1,2^* and Yanlian Yang^1,2^*

^1^CAS Key Laboratory of Standardization and Measurement for Nanotechnology, CAS Key Laboratory of Biological Effects of Nanomaterials and Nanosafety, CAS Center for Excellence in Nanoscience, National Center for Nanoscience and Technology, Beijing, China

^2^University of Chinese Academy of Sciences, Beijing, China

*** Correspondence:**Prof. Yanlian Yang

Email: yangyl@nanoctr.cn


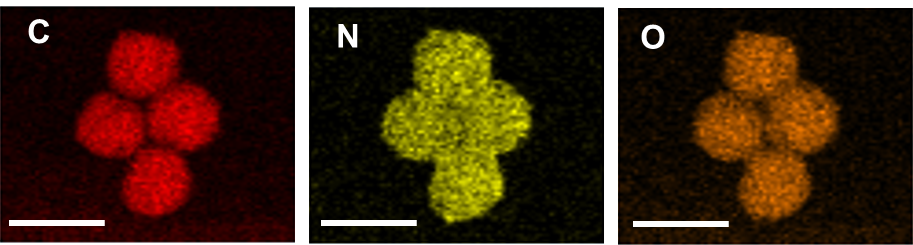


**Supplementary Figure 1.** HAADF-STEM image and elemental (C, N, O )mapping of the FeMOF (scale bar: 100 nm)


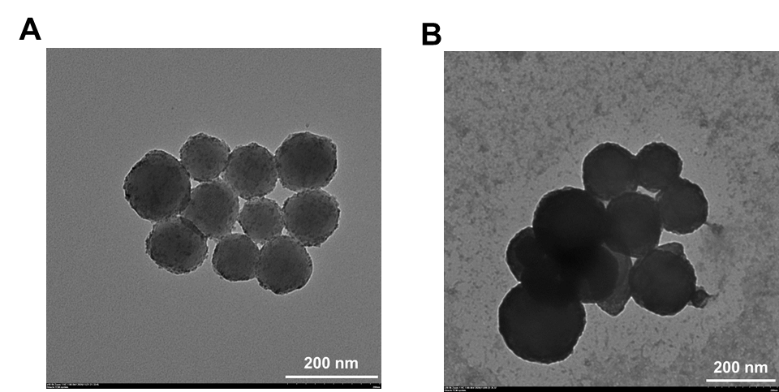


**Supplementary Figure 2.** (A) TEM image of FeMOF@HA. (B) TEM image of PyroFPSH.


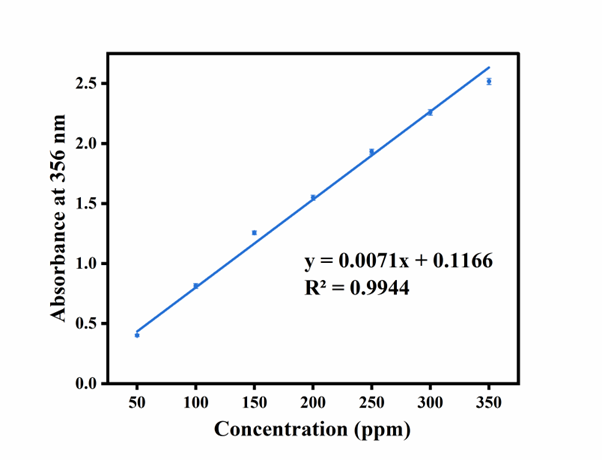


**Supplementary Figure 3.** Standard absorbance curve of different concentrations of SAS at 356nm.





**Supplementary Figure 4.** Drug release of the PyroFPSH.


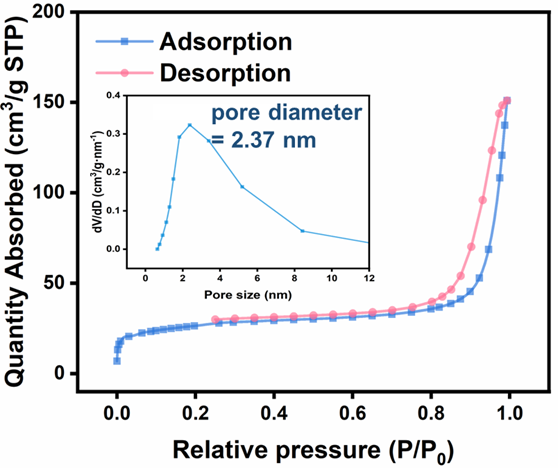


**Supplementary Figure 5.** N_2_ Adsorption-Desorption Isotherm of FeMOF


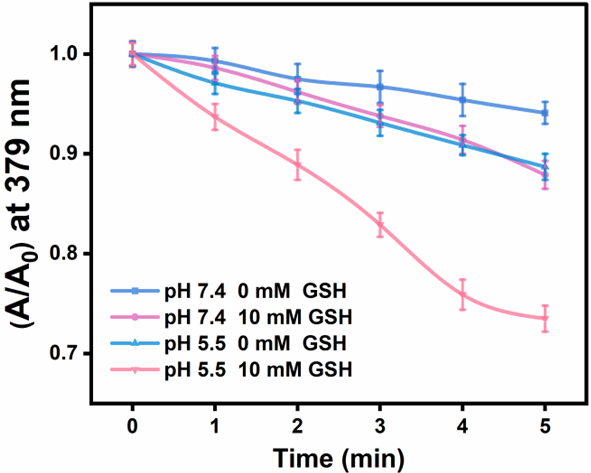


**Supplementary Figure 6.** Photodynamic effect of FeMOF@HA under Different Treatment Conditions within 5 minutes.

**
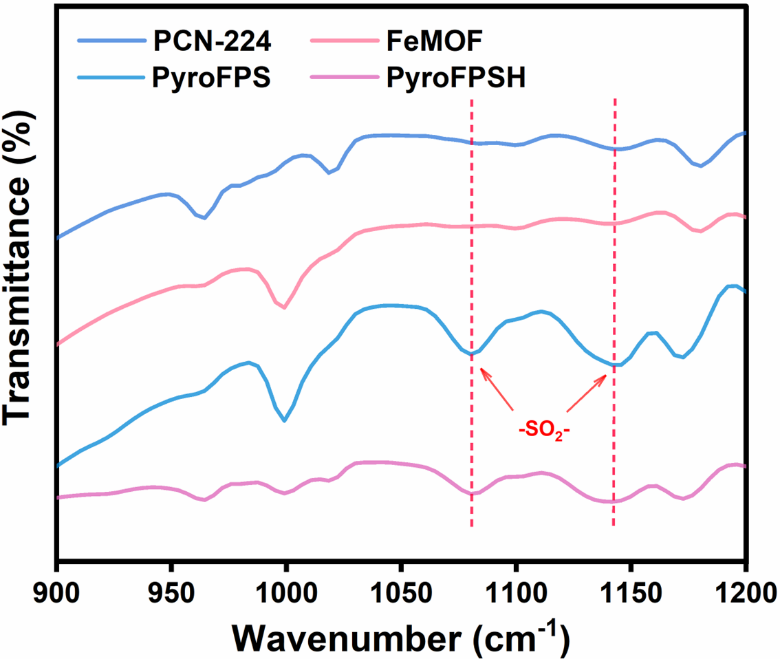
**

**Supplementary Figure 7.** FT-IR spectroscopy of -SO_2_-.


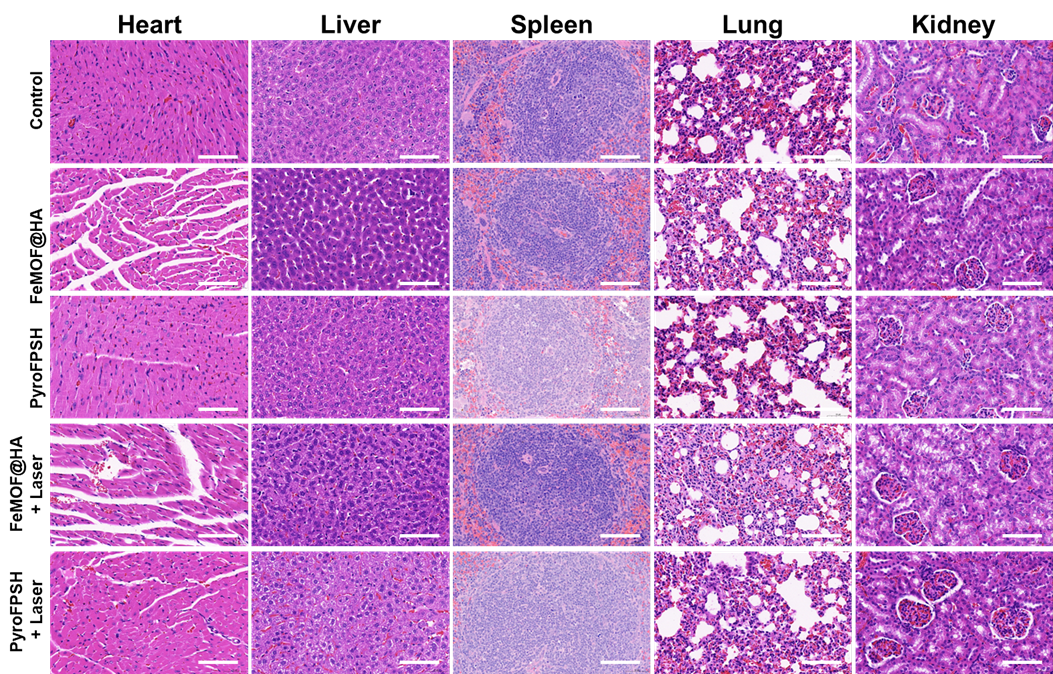


**Supplementary Figure 8.** The H&E staining of main organs (heart, liver, spleen, lung, and kidney) after different treatments (on day 14) (Scale bar: 50 μm).


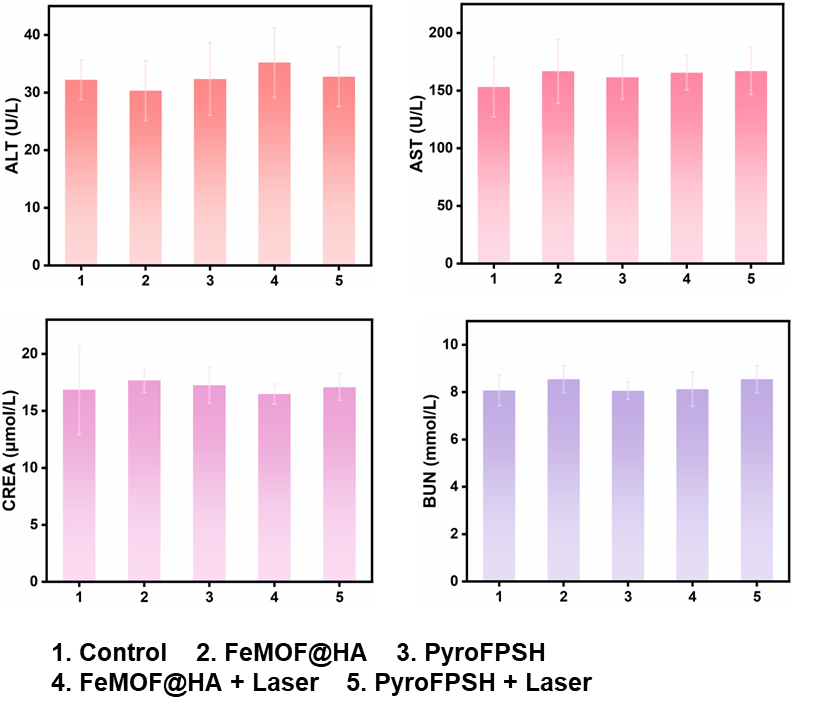


**Supplementary Figure 9.** Blood biochemistry analysis of healthy mice (Control) or mice treated. The results showed the mean and standard deviations of AST, ALT, CREA and BUN.
